# Estimating the Counterfactual Impact of Conservation Programs on Land Cover Outcomes: The Role of Matching and Panel Regression Techniques

**DOI:** 10.1371/journal.pone.0141380

**Published:** 2015-10-26

**Authors:** Kelly W. Jones, David J. Lewis

**Affiliations:** 1 Department of Human Dimensions of Natural Resources, Colorado State University, Fort Collins, Colorado, United States of America; 2 Department of Applied Economics, Oregon State University, Corvallis, Oregon, United States of America; University of Sydney, AUSTRALIA

## Abstract

Deforestation and conversion of native habitats continues to be the leading driver of biodiversity and ecosystem service loss. A number of conservation policies and programs are implemented—from protected areas to payments for ecosystem services (PES)—to deter these losses. Currently, empirical evidence on whether these approaches stop or slow land cover change is lacking, but there is increasing interest in conducting rigorous, counterfactual impact evaluations, especially for many new conservation approaches, such as PES and REDD, which emphasize additionality. In addition, several new, globally available and free high-resolution remote sensing datasets have increased the ease of carrying out an impact evaluation on land cover change outcomes. While the number of conservation evaluations utilizing ‘matching’ to construct a valid control group is increasing, the majority of these studies use simple differences in means or linear cross-sectional regression to estimate the impact of the conservation program using this matched sample, with relatively few utilizing fixed effects panel methods—an alternative estimation method that relies on temporal variation in the data. In this paper we compare the advantages and limitations of (1) matching to construct the control group combined with differences in means and cross-sectional regression, which control for observable forms of bias in program evaluation, to (2) fixed effects panel methods, which control for observable and time-invariant unobservable forms of bias, with and without matching to create the control group. We then use these four approaches to estimate forest cover outcomes for two conservation programs: a PES program in Northeastern Ecuador and strict protected areas in European Russia. In the Russia case we find statistically significant differences across estimators—due to the presence of unobservable bias—that lead to differences in conclusions about effectiveness. The Ecuador case illustrates that if time-invariant unobservables are not present, matching combined with differences in means or cross-sectional regression leads to similar estimates of program effectiveness as matching combined with fixed effects panel regression. These results highlight the importance of considering observable and unobservable forms of bias and the methodological assumptions across estimators when designing an impact evaluation of conservation programs.

## Introduction

Land cover change continues to be a leading cause of biodiversity and ecosystem service loss. There is relatively weak empirical evidence on how well conservation policies and programs—such as protected areas or payments for ecosystem services (PES)—slow or halt these land cover changes. However, the conservation field has started to emphasize the need for more rigorous evaluation of conservation approaches [1,2]. Evaluation is fairly common in the medical sciences, and increasingly in rural development and agricultural programs, and the attention in conservation is especially relevant for the new wave of conditional, incentive-based approaches that fall under the term PES, as well as the UN’s proposed Reducing Emissions from Deforestation and Degradation (REDD) program, which both emphasize additionality of land cover outcomes. While there are alternative approaches to evaluation, the use of quasi-experimental designs that establish a counterfactual outcome in order to evaluate effectiveness of a program or policy have arguably become some of the most promoted methods [1,3,4].

Quasi-experimental approaches are designed to correct for the fact that conservation programs are not randomly allocated across the landscape. This non-random allocation of where a conservation program is targeted and who enrolls in a conservation program influences the estimate of changes in land cover outcomes; this means that conventional methods of comparing changes, such as simple linear cross-sectional regression or differences in means tests, can be biased [5]. A clear example of this non-random targeting is illustrated by the placement of protected areas: most protected areas are located in places unsuitable for other economic activities, so much so that they are often given the nickname ‘rock and ice’ [6]. This remoteness reduces the impact that most protected areas have on preventing deforestation because they are much less likely to have forest cover change in the first place; of course, this does not account for any future benefits that protecting that forest today might have. One of the first studies to highlight the magnitude of the bias that arises when the non-random placement of conservation approaches is ignored was a study of the impact of Costa Rica’s protected areas on deforestation; the researchers found that cross-sectional regressions that ignore the non-random placement of protected areas overestimated the impact of parks by as much as 65% [7].

To counter these biases, quasi-experimental methods rely on the construction of a valid ‘control’ group to estimate the impact of the conservation program or policy (the ‘treatment’), where the control group is made up of observations that did not receive the conservation program. While some quasi-experimental designs utilize program rules to create the control group—for example, regression discontinuity designs and instrumental variables—the more common approaches in the conservation literature are matching and fixed effects panel methods. Matching is a statistical approach that constructs a counterfactual group based on observable variables thought to influence receiving the treatment and the outcome of interest [3]. The researcher constructs a control group that is as similar as possible to the treatment group—similarity is based on what data can be collected and is tested by comparing average values of covariates. With the matched sample of treatment and control observations, the researcher can then estimate the impact of the program using a variety of estimators, but the most common methods include a simple t-test on difference in means, or cross-sectional regression. Since the evaluation of protected area effectiveness in Costa Rica [7], the combination of matching with difference in means or cross-sectional regression has been used in a number of conservation evaluations of protected area effectiveness [8–28], and less extensively, in evaluations of PES, decentralization, land tenure, land zoning, and integrated conservation and development programs on land cover outcomes [28–38]. Reviews of conservation evaluations that use matching can be found in [39–41].

A second quasi-experimental approach to impact evaluation is to use fixed effects panel methods. This approach assumes that where a conservation program is targeted or who enrolls in it, as well as the outcome of interest, is based on observable *and* unobservable variables [3]. Panel data—data with multiple years of observation on the same cross-sectional units—must be available before and after the conservation program is implemented for the method to be used. By collecting data over time on the same observation, any time-invariant unobservable covariate is controlled through the use of fixed effects for each cross-sectional unit; the ability to control for limited forms of unobservable bias is the main advantage over cross-sectional methods. Fixed effects panel methods are a generalization of the DID method in program evaluation, where the latter use aggregate data [42]. In program evaluation, this method is sometimes referred to as a ‘before-after-control-intervention’ design. Fixed effects estimation, or DID, can be used with the full sample of treatment and control observations, or after treatment observations are ‘matched’ to a more similar control group.

In addition to controlling for time-invariant unobservables, fixed effects can be advantageous to estimation if data on observable time-invariant covariates are difficult to obtain. For example, it can be difficult to find local datasets on soil quality or rainfall that are of the same resolution as land cover data—even though coarser globally available datasets may exit. If these characteristics influence the likelihood of a conservation program or land cover change outcome, then in a fixed effects panel model they would be implicitly controlled for through the fixed effects for each cross-sectional unit. Parcel fixed effects capture all time-invariant parcel factors—observed and unobserved—that affect land cover change. In a cross-sectional study, all unobserved time-invariant parcel variables would be omitted from estimation. Similarly, if data correspond to a specific landowner, the fixed effects would control for any time-invariant household motivations to participate in the conservation program or to deforest the land. If these unobservable variables are not important (i.e. correlated with conservation variables), then cross-sectional and fixed effects methods will provide similar estimates of the impact of conservation programs on land cover outcomes. But, if they are important, then cross-sectional methods will lead to biased results, and fixed effects will move us closer to the ‘true’ estimate of impact. A comparison of experimental with quasi-experimental impact estimates for a water conservation program found that combining matching with fixed effects panel regression comes closest to replicating experimental results [43], suggesting that this might be the most robust way to estimate the impact of a conservation program when randomization is not possible. Yet, only a handful of studies that we know of use matching combined with DID or fixed effects panel methods to estimate the impacts of conservation programs on land cover outcomes [27,28,30,31,33,34].

The purpose of this paper is to highlight the potential of using fixed effects panel methods to control for time-invariant unobservables in impact evaluations of conservation programs on land cover change outcomes. There are a number of good overview papers on using quasi-experimental impact evaluation designs and the use of matching to construct a valid control group in the conservation field [1,3,44–46], but none of these emphasize the use of fixed effects methods or compare and contrast panel methods with cross-sectional analysis. Our focus on how to use fixed effects to evaluate conservation programs is particularly important given the development of new global panel datasets on forest loss; for example, the development of a global database that tracks forest status on 30-meter by 30-meter pixels annually beginning in 2000 [47]. These data would provide access to the outcome variable of interest in many conservation evaluations—land cover change. Paired with the fact that many places where we want to do impact evaluations are data constrained, the use of fixed effects methods could be a relatively easy and more robust method of conducting an evaluation of the impact of conservation policies and programs on land cover change outcomes versus cross-sectional methods, since they can control for observable and time-invariant unobservable sources of bias.

After briefly reviewing quasi-experimental impact evaluation methods in Section 2, we compare impact estimates using matching to construct a control group combined with difference in means and cross-sectional regression to fixed effects regression with and without matching to create the control group for two conservation programs: a national PES program in Northeastern Ecuador and strict protected areas in European Russia. We discuss whether these methods lead to differences in statistical significance or the magnitude of the conservation outcome—conclusions that affect the policy implications of these conservation programs on land cover change. Our overall goal is to provide additional guidance on how the choice of quasi-experimental design can affect estimated program outcomes and suggest when attention to observable versus unobservable variables is important in estimating treatment effects.

## Quasi-Experimental Impact Evaluation Methods

### Matching

A major emphasis in the conservation evaluation literature has been the non-random placement of many conservation interventions, which lead to selection bias in estimation [44]. As a result, the use of matching to construct a control group that is ‘as similar as possible’ to the observations that receive the conservation program has been emphasized. Using observable data, treatment observations are ‘matched’ to their most similar control observations and then this new sample is used to estimate the effect of the conservation policy or program on the land cover outcome [5,44,48–50]. The process of finding the most similar control observations and then estimating the treatment effect with this new sample reduces the bias present in estimating the treatment effect using all the possible control observations, many of which are very different from the places or households that receive conservation programs. There are a number of matching algorithms and estimators available [51–53]. Some of the more popular matching algorithms include propensity score matching and covariate matching. In propensity score matching, the researcher estimates a probability of receiving the treatment for each observation, and then matches units in the treatment group to those outside the program that have the closest propensity score [54]. In covariate matching, treatment observations are matched to those that did not receive the program based on individual covariates using a multivariate distance metric [53]. Many matching algorithms can be implemented using pre-programmed codes in Stata or R. The new, matched, sample of treatment and control observations can then be used to calculate the treatment effect with a variety of estimators. The simplest method would be to take the average of the differences in means across the matched treatment and control observations.

### Combining Matching with Cross-Sectional Regression

It is highly recommended that matching to produce the control group be combined with post-matching cross-sectional regression to estimate the treatment effect [5,50,54]. This is because, in many cases, the matching algorithm will not have balanced all of the observable covariates across treatment and control observations, and so additional balancing is necessary. Some estimators have built-in bias-adjustment options that automatically implement a post-matching cross-sectional adjustment [53].

The major assumption made when using a difference in means test or cross-sectional regression after matching the sample is that there are no unobserved characteristics associated both with potential outcomes and treatment that could bias the estimation; this is known as the unconfoundedness assumption [5]. While there are options to test the sensitivity of results from matching estimators to hidden bias—through tests such as Rosenbuam bounds—these tests cannot explicitly tell whether hidden bias is actually present or not [54].

### Fixed Effects Panel Regression

As applied to the land-cover change setting, fixed effects panel regression uses repeated temporal observations of land cover change on the same plot or parcel of land. Rather than relying on the statistical construction of a control group as in matching, panel techniques use the temporal dynamics of the data—observing the treatment and control observations before and after treatment—along with cross-sectional variation in treatment status across plots to construct the counterfactual outcomes. The key dependent variable indicates land cover change (LCC) for plot *i* in time *t*, denoted *LCC*_*it*_. For example, suppose plot *i* was deforested in time *t* = 4 over a 5-year panel. Then, *LCC*_*i*4_ = 1 while *LCC*_*it*_ = 0 for *t* = 1, 2, 3, and the plot is dropped in *t* = 5 since the parcel is no longer forested (assuming binary measure of forest/non-forest). A generic fixed effect panel regression equation is constructed as follows:

$$LCC_{it}=\alpha +\beta_{1}X_{it}+\beta_{2}G_{i}+\beta_{3}C_{it}+\beta_{4}Y_{t}+a_{i}+\varepsilon_{it},$$ (1)

Where *X*_*it*_ denotes time-varying covariates of land cover change for plot *i* (e.g. distance to forest edge), *G*_*i*_ denotes time-invariant covariates of land cover change (e.g. slope), *C*_*it*_ indicates the time-varying conservation status of plot *i*, *Y*_*t*_ indicates time fixed effects, and *a*_*i*_ + *ε*_*it*_ is the composite unobservable. In panel data analyses, the model unobservable is decomposed into a time-invariant component (*a*_*i*_) and a time-varying component (*ε*_*it*_). The primary goal of estimation is to obtain a consistent estimate of the treatment effect of conservation, *β*_3_.

If all observable covariates (*X*_*it*_, *G*_*i*_, *C*_*it*_) are independent of the composite unobservable (*a*_i_ + *ε*_*it*_), then cross-sectional regression will generate a consistent estimate of *β*_3_. Omitted variable bias arises most directly if conservation status *C*_*it*_ is correlated with (*a*_i_ + *ε*_*it*_). For example, suppose conservation is targeted at forest types with low timber value but data do not exist on forest type. Then since forest types are likely time-invariant over reasonably short panels, and since forest types of low timber value are less likely to get harvested than types of high timber value, *C*_*it*_ would be correlated with *a*_i_ and cross-sectional estimation of *β*_3_ would be biased. The conservation status of plot *i* is confounded by the unobserved type of forest stand that exists on plot *i*. If *C*_*it*_ is not correlated with (*a*_i_ + *ε*_*it*_), then omitted variable bias could still arise indirectly if either of the observable covariates (*X*_*it*_, *G*_*i*_) are correlated with both the composite unobservable (*a*_i_ + *ε*_*it*_) and with the primary conservation treatment variable *C*_*it*_.

Estimating the time-invariant component (*a*_*i*_) in Eq 1 as a fixed effect aims to reduce the degree of omitted variable bias described in the previous paragraph by exploiting temporal variation in conservation status *C*_*it*_ within plots of land. In particular, fixed effects estimation breaks any correlation between observable time-varying variables (*X*_*it*_, *C*_*it*_) and the time-invariant unobservable (*a*_*i*_) by transforming the data in two steps. First, for each plot *i*, average Eq (1) over time:

$$\bar{LCC_{i}}=\alpha +\beta_{1}\bar{X_{i}}+\beta_{2}G_{i}+\beta_{3}\bar{C_{i}}+\beta_{4}{\bar{Y}}_{t}+a_{i}+\bar{\varepsilon_{i}},$$ (2)

where $\bar{LCC_{i}}=\;T^{-1}\Sigma_{t=1}^{T}LCC_{it}$, and so on for the other variables. Notice that the time-average of *G*_*i*_ and *a*_*i*_ remains fixed since these variables are time-invariant. Second, subtracting Eq (2) from Eq (1) yields the classic “within” estimator in panel regression:

$$LCC_{it}-\bar{LCC_{i}}=\beta_{1}\left(X_{it}-\bar{X_{i}}\right)+\beta_{3}\left(C_{it}-\bar{C_{i}}\right)+\beta_{4}(Y_{t}-{\bar{Y}}_{t})+\left(\varepsilon_{it}-\bar{\varepsilon_{i}}\right).$$ (3)

Notice two main components of Eq (3). First, the parameters on the time-varying covariates (*β*_1_, *β*_3_, *β*_4_) are preserved in the differencing, and so least-squares regression of the “within-transformed” data in Eq (3) enables estimation of the primary treatment effect *β*_3_. Second, the time-invariant observable (*G*_*i*_) and the time-invariant unobservable (*a*_*i*_) are differenced out of Eq (3), and so consistent estimation on the within-transformed data in Eq (3) does not require the researcher to assume *any* independence between the time-varying covariates (*X*_*it*_, *C*_*it*_) and the time-invariant unobservable (*a*_*i*_). Identical estimates are produced by estimating Eq (1) with a dummy variable for each plot, though the within estimator in Eq (3) is preferred as a means of avoiding estimation of potentially many thousands of dummy variable parameters. Time fixed effects, *Y*_*t*_, on the other hand, are estimated as dummy variable parameters. Their inclusion controls for unobservables that differ across time but not observations, for example, national policy changes or global commodity prices.

For fixed effects to be a valid estimation strategy, parallel paths, or trends, must exist prior to treatment [42]. While this assumption cannot be directly tested, a graph of the temporal trends in outcomes for treatment and control observations before and after treatment can provide some visual assurances: we want to observe land cover outcomes on similar—or parallel—trajectories across treatment and control groups prior to the program. A more robust check is to statistically test whether there is a difference in pre-treatment outcomes by introducing an interaction term between *C*_*it*_ and *Y*_*t*_. If the coefficient is not statistically significant in years prior to treatment then the parallel paths condition is more likely to hold.

### Combining Matching with Fixed Effects Panel Regression

While fixed effects panel methods can be used on the full sample of treatment and control observations, using temporal variation to construct the control group, it can also be combined with matching. In this case matching is first used to “pre-process” the data to find the best set, or most similar set, of treatment and control observations before fixed effects regression is used to estimate the treatment effect. Estimating fixed effects regression on a matched sample can reduce omitted variables bias in the following sense. Suppose an element of the time-varying covariate vector *X*_*it*_ is correlated with both a plot’s conservation status *C*_*it*_ and with the time-varying unobservable *ε*_*it*_. Then the regression estimate of the treatment effect *β*_3_ will be biased because *C*_*it*_ will be indirectly correlated with *ε*_*it*_ through *X*_*it*_. Fixed effects estimation will not help reduce this bias since none of these time varying components (*X*_*it*_, *C*_*it*_, *ε*_*it*_) are differenced out. A matched sample helps reduce bias in this situation because matching reduces correlation between the observables *C*_*it*_ and *X*_*it*_ by making treated plots (*C*_*it*_ = 1) similar to control plots (*C*_*it*_ = 0) in their observed values of *X*_*it*_. Thus, fixed effects regression on the matched sample is less sensitive to correct specification of *X*_*it*_ in the model. Even with a dataset where there are no time-varying observables (*X*_*it*_) other than conservation treatment (*C*_*it*_), matching can still improve estimates from fixed effects regression. One reason is that the linear fixed effects model assumes that all landowners have a common response to the conservation treatment (*β*_3_)–and this assumption is more plausible when treated and untreated landowners have similar levels of fixed characteristics rather than very different levels [43].

## Methods

### Conservation Programs

Table 1 summarizes the two conservation programs we analyze in our comparison of the impact evaluation methods discussed above: (1) PES in Northeastern Ecuador and (2) strict protected areas in European Russia. These two programs cover the more common conservation approaches for which impact evaluation is being used and illustrate these methods across both continuous (Ecuador-PES example) and binary measures (Russia-Protected Areas example) of forest cover outcomes. No specific permissions were required to conduct these analyses and all data were acquired from publicly available data sources.

**Table 1:** Summary of Conservation Programs.

	Ecuador-PES	Russia-Protected Areas
Definition and number of treatment observations	Individual landowner parcels that enrolled in PES N = 63	Random sample of pixels within four protected areas N = 5,019
Definition and number of control observations	Individual landowner parcels that did not enroll in PES N = 450	Random sample of pixels outside of protected areas N = 20,670
Unit of analysis	Household Parcel	Pixel
Years of land cover data available	Annual forest cover measures between 2004–2013	1990 to 2010 in 5-year increments
Year of treatment	2010	1990–1995
Outcome of interest (Continuous/Binary)	Percent change in forest cover (Continuous)	Change from forest to non-forest (Binary)
Observable covariates	Baseline deforestation Parcel size Distance to roads, towns, major rivers, oil wells	Elevation Slope Distance to forest edge, towns, capital city, roads
Potential unobservable covariates	Household demographics Conservation motivations Agricultural suitability	Soil quality Rainfall Tree species/forest type

#### Ecuador-PES

In Ecuador, the study region is about 600 sq km around the northwestern border of Cuyabeno Faunal Wildlife Reserve, a protected area in the northeastern Ecuadorian Amazon. The Cuyabeno Reserve was officially created in 1979 and deforestation by smallholders living adjacent to the boundaries of the reserve has been a continuous conservation threat. Tenure insecurity is commonly cited as a driver of deforestation in the region [55]. In 2009, a nationally sponsored land titling effort resulted in the acquisition of private individual titles for smallholders that were living adjacent to the reserve; in all cases, these households occupied their land since the late 1970s or early 1980s but had not received formal title. In 2008, Ecuador implemented a national PES program, known as Socio Bosque [56]. The program targets forested lands that include a combination of multiple ecosystem service benefits, risk of deforestation, and populations with high degrees of social marginality. To be eligible to participate, a property must have a formal land title, meaning that smallholders around Cuyabeño Reserve became eligible for the program after they received their formal land titles in 2009; 63 households enrolled in the program in 2010.

Since PES is a household-level program, the ideal unit of analysis is the household parcel [44]; spatial boundaries of household parcels are available for this study area from a cadastral survey conducted by the Government of Ecuador. If this information were not available then it would be difficult to rigorously evaluate the impact without collecting property boundaries using GPS, even though some evaluations of PES have used pixels, or grid cells, for evaluation [36], these do not represent the decision-making unit. In addition to parcel-level information for households that enrolled in PES, we also know which households did not enroll, providing us with a group of control observations that are likely to share at least some characteristics with the treatment observations given the small study area. To further refine this control group, we limit the pool of potential controls to titled smallholder parcels that are within the same pre-cooperatives as the households that enrolled in PES. Pre-cooperatives are self-organized groups of smallholders that precede the land titling campaign, and represent a spatially contiguous group of households that migrated to the area in a similar time period. This gives us 450 households as potential controls. Defining control observations as parcels that are near enrolled households is similar to the strategy used in [31], but a stricter definition of PES controls would be to use households that applied but were rejected from the PES program, or households that applied in later years [29,30]; neither of these options exist for these data.

We measure the impact of this PES program on reduced deforestation rates. We use a globally available forest/non-forest product with resolution of 30 meter by 30 meter to measure deforestation [46]. While this dataset provides pixel-level information on forest cover, we aggregate it to household parcels, providing a continuous measure of forest cover loss per parcel between 2004 and 2013. While this land cover product provides data as early as 2001, we excluded these early years because our test of parallel trends detected differences in 2003 across our two groups. Thus, we can estimate a treatment effect for the impact of PES on reducing deforestation between 2011–2013, controlling for pre-treatment deforestation rates in 2004–2010.

PES is a voluntary program, and household-level characteristics, such as age, income, and prior conservation motivations, as well as parcel-level characteristics, such as size, agricultural suitability, and accessibility, are expected to influence a smallholder’s decision to participate in the program [57,58]. These characteristics would also influence the probability of deforestation with or without the program. Past evaluations of PES programs on land cover change have controlled for parcel size, accessibility, agricultural productivity, baseline deforestation rates, forest type, past participation in forestry programs, poverty level, and tenure [29–31,34,36,38]. In our study area there are a number of available datasets measuring parcel-level characteristics related to accessibility and parcel size, but we do not have household-level information on past participation in conservation programs or poverty. Additionally, we do not have data directly related to agricultural suitability. Thus, the observable covariates that we can control for include parcel-level information on pre-treatment deforestation rates (between the years 2004–2010), parcel size, and distance of the parcel to roads, population centers, navigable rivers, and oil wells, which control for remoteness and opportunity costs in this region. Our potential unobservable variables are related to household-level characteristics that would lead a household to enroll in the program or to deforest, such as environmental motivations, and parcel-level agricultural productivity. However, since our unit of analysis corresponds with the decision-making unit, if these variables are time-invariant unobservables, they will be controlled for with the fixed effects methods.

#### Russia-Protected Areas

In Russia, our analysis focuses on the effect of four ‘strict’ protected areas—known as zapovedniks in Russia—in temperate European Russia. This is a subset of the data used to compare the effectiveness of different types of protected areas in Russia before and after the collapse of the Soviet Union [27]. Strict protected areas are equivalent to an IUCN designation of Category I protected areas and logging and other extractive activities are prohibited [59]. Russia established a number of new protected areas following the collapse of the Soviet Union in 1991 [60], and the four parks in this analysis were established between the years 1992 and 1994. Their total area is 515 km^2^.

Within a protected area, a remote sensing pixel is a common unit of analysis [44]. Since each protected area contains thousands of pixels, referred to as plots in the remainder of the paper, we must sample to get a manageable number of observations. We sample 1% of all plots within parks that were forested in our baseline year– 1990. For control observations we randomly sample forested plots outside of protection. In Russia, all forested land is publically owned, so when sampling forest outside of parks the decision-making unit should also be the Russian Forest Service, although they do lease this land to private timber concessions. We sample four-times the number of treatment plots from areas outside of protected areas to generate the potential control group.

The outcome of interest in this case is forest disturbance for harvesting timber [61]–which varies from deforestation in the Ecuador case, since the land will eventually revert back to forest. Measures of forest disturbance come from a primary Landsat classification of forest cover change over 5-year increments from 1985 to 2010 [62]; we exclude the 1985–1990 period since forest disturbance follows different temporal trends during this period, likely due to political unrest. This primary analysis provides 30-meter resolution data on forest cover change with average accuracies greater than 90%. Forest disturbance is measured as a binary outcome, and so for each observation a value of “0” is recorded if there is no change within a 5-year period or a “1” if there is a change; a plot is removed from the dataset once change occurs so that forest disturbed on this plot—the outcome variable—is not double counted. Given the year of designation of the protected areas, pre-treatment forest disturbance is defined as the 1990–1995 time period and forest disturbance that occurs anytime between 1995 and 2010 is our outcome variable.

The majority of published evaluations of protected area effectiveness focus on tropical regions—where land cover change is driven by agriculture. In these studies, data is collected on locational and biophysical characteristics of the pixel or grid cell, such as slope, elevation, accessibility, agricultural suitability, and forest type [7–23]. Forest disturbance in European Russia is increasingly correlated with profit-maximization behaviors that factor in transportation costs and opportunity costs of the land for timber harvesting [63]. Thus, for both protected area designation and forest disturbance in Russia we control for biophysical characteristics of the plot and its location. The observable covariates include elevation, slope, and distance of the plot to the forest edge, closest town, Moscow, and closest road. There are important differences between the type of observable data that can be controlled for with binary and continuous outcome measures of land cover change worth highlighting here. With continuous measures a baseline measure of land cover change can be controlled for (e.g., in Ecuador, baseline deforestation rates). With binary data baseline rates cannot be included, but measures such as distance to forest edge, which would measure accessibility, can be included (e.g., Russian covariates).

Since the Forest Service manages forested plots in Russia, potential unobservable covariates are less likely to be related to differences in decision-making units, unless “controls” are in areas of private timber concessions. The more likely source of omitted time-invariant covariates, however, is if there are unobservable biophysical characteristics that influence protected area status and or forest disturbance. For example, soil quality, rainfall or type of tree species might influence conservation decisions if protection is targeted at specific ecological species, or might influence forest disturbance rates if some species are preferred for timber. In Russia, these datasets are not available and globally available datasets are typically at 1-square kilometer resolution.

### Empirical Methods

**Fig 1:**
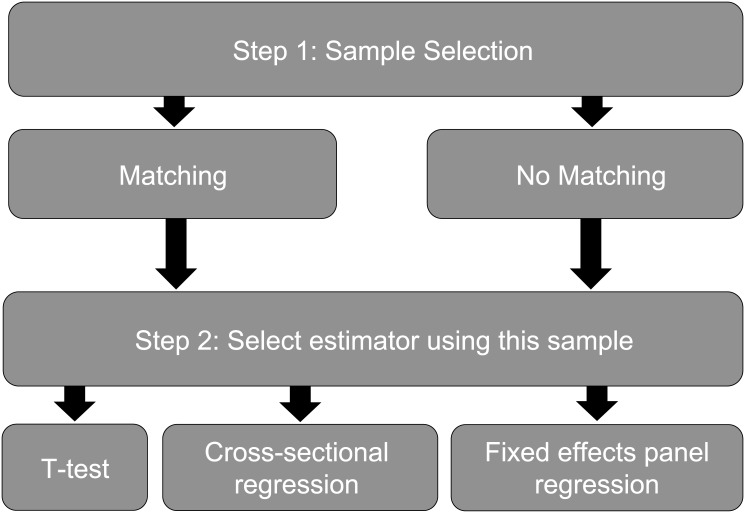
Illustration of Sample Selection and Estimator Choice in this Study.

As discussed above, matching is often used to construct a valid control group in program evaluation, and is then combined with several post-matching estimators to get the treatment effect. Fixed effects panel methods can be used with or without matching to construct the control group. Effectively, this gives six combinations across the sample selection process and the estimation process for program evaluation (Fig 1). However, a number of sources have illustrated why ‘no matching’ combined with simple differences in means or cross-sectional regression are biased in conservation program evaluation [7,43,45]. The more common methods seen today in the conservation evaluation literature include combining ‘matching’ with either simple differences in means or cross-sectional regression. Thus, to compare these more common methods to fixed effects panel methods, we estimate treatment effects for the two conservation programs described above using the following approaches: (1) matching plus differences in means, (2) matching plus cross-sectional regression, (3) ‘no matching’ plus fixed effects panel regression, and (4) matching plus fixed effects panel regression.

Our analysis of the Ecuador-PES program uses a limited continuous variable which is never negative and includes a non-trivial 37% of parcels with a zero deforestation rate, while our analysis of Russian parks models a binary dependent variable for each plot which equals one if the plot is deforested and zero otherwise. Alternative non-linear maximum likelihood estimators for such limited dependent variables include Tobit and Probit models. We choose to use linear ordinary least squares regression rather than non-linear estimation approaches for the following reason: our primary interest is in obtaining a consistent estimate of the treatment effect of conservation—known as a marginal or partial effect—rather than predicting the level of deforestation. Thus, under similar identifying assumptions, linear ordinary least squares typically generates similar marginal effects as non-linear methods and has the advantage of being able to include fixed effects in a far simpler fashion—and with fewer assumptions—than non-linear methods [42]. Fixed effects cannot typically be used in most non-linear methods due to the incidental parameters problem [64].

#### Matching

We match treatment to control observations using propensity score matching [54]. The propensity score is a measure of the probability of an observation receiving the conservation program. Since both conservation programs have binary treatments, the probability of receiving the program is estimated with a Logit model specified on the set of observable pre-treatment covariates thought to influence treatment assignment and the outcome of interest identified in Table 1 (regression output in Table A in S1 and S2 Tables). Using the estimated propensity scores, each treatment observation is then paired with the ‘best’ control observation based on one-to-one nearest neighbor matching without replacement following [65]. To ensure ‘good’ matches the distance between propensity scores is limited by using a caliper size of a quarter of the standard deviation of the estimated propensity score as recommended by [54]. In the case of propensity score matching, the probability of receiving treatment varies between ‘0’ and ‘1’, and the caliper limits the distance that the algorithm searches to find an appropriate control observation for the treatment unit (e.g., a caliper of 0.2 would limit the search to two-tenths). Our choice of matching algorithm is motivated by its popularity in the literature and the ease at which the matched sample can be exported for use in cross-sectional or fixed effects panel regression. While alternative matching algorithms might yield a slightly different sample of treatment and control observations, post-matching estimations of treatment effects with this sample would yield similar relative differences across findings to what we present below since it is the presence of time-invariant unobservables that potentially lead to bias in cross-sectional estimation.

With our matched sample, treatment effects are estimated under three estimators. The difference in means computes the average difference in outcomes between each treatment and control observation. For cross-sectional estimation, the matched sample is used in a linear ordinary least squares regression, controlling for the same observable covariates included in the match. The fixed effects panel estimator is described below.

#### Fixed Effects Panel Regression

First, we estimate a fixed effects panel regression for each conservation program using all treatment and control observations, i.e., ‘no matching’. We could include the full list of observable covariates in Table 1, but since most observable covariates are time-invariant—with the exception of distance to forest edge in Russia—they fall out of the differencing equation, that is, they are not explicitly estimated in the regression output. However, since we have land cover change data before and after the conservation programs, *β*_3_ in Eq 1 can be estimated.

Second, we use matching to pre-process the data as described above, and then use this trimmed sample to estimate *β*_3_ in Eq 1. When matching the Ecuador-PES data, baseline deforestation is defined as years 2004–2006; these years are then omitted from the fixed effects regression.

#### Standard Errors

For cross-sectional and fixed effects regressions we use cluster robust standard errors to control for spatial autocorrelation. Clustering standard errors relaxes the assumption of no correlation across observations within the spatial unit used for clustering. For Ecuador we cluster at the informal administrative unit known as pre-cooperatives. In Russia we cluster at a political unit similar to counties in the United States. In addition to being robust against any form of spatial correlation within clusters, cluster robust standard errors also control for general forms of serial correlation and heteroskedasticity in panel regressions [66].

#### Similarities and Differences across Estimators

These methods ensure two things that aid in comparison across estimators. First, across three of the estimators, propensity score matching is used for sample selection, ensuring that the same treatment and control observations are compared. The only difference is that post-matching, one estimator controls for limited forms of omitted variables bias (i.e., fixed effects) and two do not (i.e., t-tests and cross-sectional regression). While fixed effects without matching necessarily uses all of the treatment and control observations, the analysis of these data is exactly the same as that in fixed effects regression following matching, allowing us to compare the effect of using matching to pre-process the data on estimated impacts. Secondly, we are able to estimate similar standard errors—cluster robust standard errors—for each method of analysis except for matching with differences in means. This ensures that our standard errors are robust to many known problems associated with land cover panel data (i.e., heteroskedasticity, spatial correlation within clusters).

#### Goodness of Fit Checks

We check whether there is improvement in covariate distribution after matching treatment to control observations and whether there is overlap of propensity score values for treatment and control observations. For the former, a t-test can be used, but differences can be skewed by sample size. The normalized difference in means is preferable over the t-statistic when there are large differences in sample size [5]. We report both since there is no statistical test of significance for normalized difference in means; although a general rule of thumb is that differences larger than 0.25 is indicative of sensitivity of estimates to functional form in linear regression [5]. Overlap of propensity scores can be plotted using a histogram.

For fixed effects regression we check the parallel paths assumption. We do this by graphing trends and by introducing an interaction term to Eq 1 and estimating whether there is any difference in forest outcomes prior to treatment.

## Results and Discussion

### Ecuador-PES

**Fig 2:**
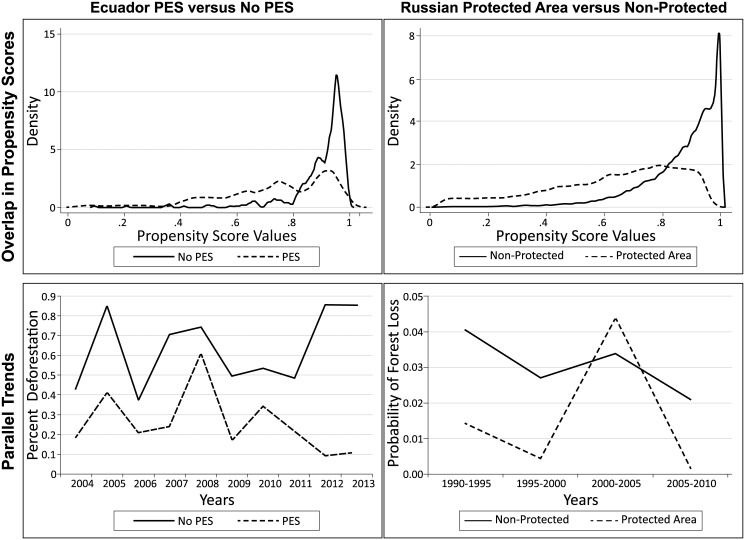
Goodness of Fit Tests. Overlap in propensity scores is calculated in Stata 13; it shows the distribution of propensity scores across treatment and control observations. Parallel trends are graphed for both treatment and control groups before matching. A test for statistical differences in trends was estimated using fixed effects regression. After matching, trends become more similar, and are not shown here.

Households that enrolled in Ecuador’s national PES program differ across observable characteristics from households that did not enroll (Table 2). On average, they have larger parcels of land, are farther from roads and towns, and have lower rates of deforestation before enrolling in the program. Propensity score matching does significantly improve the covariate balance between these two groups as indicated by the t-tests and normalized differences in means following matching. There is good overlap in estimated propensity score values between Ecuador-PES and Ecuador-non-PES households despite these differences in covariates (Fig 2). The graph of parallel trends using the full sample also illustrates the different pre-treatment deforestation rates of these two groups; despite a lower baseline deforestation rate, the temporal patterns of both groups appear to trend in similar patterns before treatment suggesting that fixed effects estimation is appropriate to use. When statistically tested using fixed effects regression, we find no differences in pre-treatment forest outcomes across these two groups between 2004 and 2010.

**Table 2:** Summary Statistics for Ecuador-PES.

Variable	Ecuador- PES	Ecuador-Non-PES	T-test		Normalized Difference in Means	
			Before match	After match	Before match	After match
Post-treatment deforestation (2011–2013) (Average annual %)	0.15 (0.31)	0.73 (1.43)	7.50***	2.96***	-0.39	-0.39
Pre-treatment deforestation (2004–2010) (Average annual %)	0.31 (0.53)	0.59 (1.1)	3.365***	-0.44	-0.24	0.03
Size of parcel (sq km)	0.64 (0.25)	0.47 (0.26)	-5.37***	0.30	0.49	-0.04
Distance to closest town (km)	4.81 (3.37)	3.89 (3.03)	-2.07**	0.88	0.21	-0.12
Distance to closest road (km)	4.13 (4.22)	1.77 (2.32)	-4.35***	-0.94	0.47	0.10
Distance to closest river (km)	8.43 (2.91)	8.17 (3.35)	-0.65	-0.70	0.05	0.07
Distance to closest oil well (km)	3.90 (3.19)	2.61 (2.44)	-3.09***	-0.74	0.27	0.07
*Observations*	*63*	*450*	*513*	*112*	*513*	*112*

*p<0.1;

**p<0.05;

***p<0.01

Standard deviations in parentheses. T-tests test for differences in means assuming unequal variances. “Before matching” uses the full sample; “After matching” is based on 1-to-1 propensity score matching without replacement and limiting the maximum distance between matches with a caliper. Normalized differences in means are calculated as recommended by [5]. There is no test for statistical significance associated with normalized differences in means but a rule of thumb is that sizes larger than 0.25 can bias simple ordinary least squares regression.

**Fig 3:**
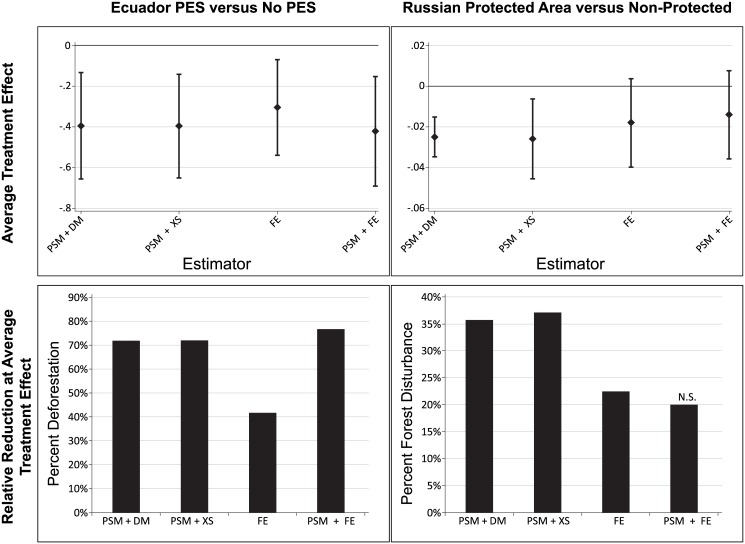
Treatment Effect Distribution and Relative Reduction in Deforestation (Ecuador) and Forest Disturbance (Russia). Box plots show the average treatment effects and 95% confidence intervals (from Table 3). For estimators that use matching, the relative reduction in forest cover change is calculated as the estimated average treatment effect (Table 3) divided by the deforestation rate (Ecuador-PES) or the probability of forest cover change (Russia-Protected Areas) in the matched control observations after treatment. When matching is not used, the deforestation rate or probability of forest cover change in the full set of control observations is used. As an example, the relative effect for Ecuador-PES under propensity score matching and differences in means is calculated as 0.40/0.55 = 0.72. (The 0.55 is not reported in the tables but comes from calculating average deforestation in 2011–2013 from the matched control units.) For the same estimation strategy and Russia-Protected Areas, the relative reduction would be calculated as 0.03/0.07; again 0.07 is not reported in the tables. This gives the relative reduction in forest cover change that can be attributed on average to the conservation program. The four estimators are abbreviated as follows: PSM +DM: propensity score matching plus differences in means; PSM + XS: propensity score matching plus cross-sectional regression; FE: fixed effects panel regression; PSM + FE: propensity score matching plus fixed effects regression.

Using the matched sample, differences in means and cross-sectional regression both estimate a treatment effect of -0.4. This is statistically significant at the 99% level and can be interpreted as a four-tenths of a percentage point reduction in the average annual deforestation rate between 2011–2013 for parcels that enrolled in PES compared to parcels that did not enroll (Table 3). To put this in perspective, post-treatment deforestation rates on all parcels not enrolled in the PES program were an average of 0.7% per year (Table 2). For no matching and fixed effects regression, the estimated treatment effect is slightly smaller, at -0.3, but still statistically significant. Finally, matching combined with fixed effects regression yields a treatment effect of -0.4, statistically significant at the 99% level. Full regression output can be found in Tables B-D in S1 Table. We plot the coefficients in Table 3 with their 95% confidence intervals to detect the level of overlap (Fig 3). In all cases, the confidence intervals of the estimated treatment effects overlap substantially.

**Table 3:** Treatment Effects under Four Different Empirical Estimators.

	Matching + Differences in means	Matching + Cross-sectional regression	No matching + Fixed effects panel regression	Matching + Fixed effects panel regression
**Ecuador-PES**				
*Treatment effect* *(change in annual parcel-level deforestation rate)*	-0.395*** (0.133)	-0.396*** (0.130)	-0.305** (0.120)	-0.422*** (0.137)
*N*	*112*	*112*	*3*,*591*	*784*
**Russia-Protected Areas**					
*Treatment effect* *(change in probability of plot-level forest disturbance over 5 year time periods)*	-0.025*** (0.005)	-0.026** (0.010)	-0.018* (0.011)	-0.014 (0.011)
*N*	*9*,*095*	*9*,*095*	*106*,*950*	*36*,*217*

*p<0.10;

**p<0.05;

***p<0.01

Standard errors in parentheses.

To better understand the conservation implications across these estimators, the relative reduction in forest cover change at the average estimated treatment effect is presented in Fig 3. This relative reduction in forest cover is calculated by dividing the estimated treatment effects in Table 3 by the rate of forest cover change in the matched control observations after the conservation program began (i.e., post-treatment), or in the case of ‘no matching’ combined with fixed effects regression, all control observations. In the Ecuador-PES program, the matched control observations have an annual deforestation rate of 0.55% in 2011–2013; if you divide the estimated treatment effect from propensity score matching plus difference in means (-0.40) by 0.55 you get 0.72. Thus, the average annual impact of PES is an approximately 72% reduction in the deforestation rate when estimated using matching with difference in means; this represents a decrease in average annual deforestation on a parcel from 0.55 to 0.16. For fixed effects regression without matching, -0.31 is divided by post-treatment deforestation in the full sample, which is 0.73, resulting in a relative effect of 42%.

### Russia-Protected Areas

Table 4 shows summary statistics for the sample of plots within and outside of strict protected areas in European Russia. Protected plots appear more remote than unprotected plots—they are farther from the forest edge and closest road. However, they are closer to towns and the capital city, Moscow. The differences in means tests before matching find very large differences, and even after matching statistically significant differences remain. However, calculations of normalized differences in means after matching find that the differences are less than 0.1; well below the 0.25 rule of thumb. There is also overlap in propensity score values (Fig 2). In the graph of parallel trends there does appear to be similar trends in the five years prior to protected area designation (i.e., 1990–1995) (Fig 2). A formal test using regression analysis finds no statistical difference in forest disturbance in the 1990–1995 time period. There also appear to be similar trends after protected area status indicating that the treatment effect, if any, is not likely to be large. It is important to point out the relatively small change in forest disturbance over the five-year periods in this sample—rates of change are less than one-tenth of a percentage point.

**Table 4:** Summary Statistics for Russia-Protected Areas.

Variable	Russia-Protected area plots	Russia-Non-protected plots	T-test		Normalized Difference in Means	
			Before match	After match	Before match	After match
Post-treatment forest disturbance (Average 5-year change, 1995–2010) (%)	0.05 (0.22)	0.08 (0.27)	7.99***	5.06***	-0.08	-0.08
Pre-treatment forest disturbance (5-year change, 1990–1995) (%)	0.01 (0.12)	0.04 (0.20)	12.59***	N/A^a^	-0.42	N/A^a^
Distance to forest edge in 1990 (km)	0.72 (0.57)	0.31 (0.36)	-49.49***	-5.21***	0.49	0.06
Distance to closest town (km)	60.88 (24.40)	75.46 (30.88)	36.69***	1.58	-0.34	-0.03
Distance to Moscow (km)	473.99 (178.58)	517.60 (213.94)	15.19***	-5.12***	-0.16	0.09
Distance to closest road (km)	1.68 (1.31)	1.25 (1.01)	-22.32***	2.60***	0.28	-0.02
Elevation (m)	152.09 (62.23)	170.65 (45.06)	20.18***	3.74***	-0.24	-0.06
Slope (%)	1.33 (1.44)	1.42 (1.53)	3.96***	1.52	-0.03	-0.02
*Observations*	*5*,*113*	*22*,*920*	*28*,*033*	*9*,*095*	*28*,*033*	*9*,*095*

*p<0.1;

**p<0.05;

***p<0.01

Standard deviations in parentheses. T-tests test for differences in means assuming unequal variances. “Before matching” uses the full sample; “After matching” is based on 1-to-1 propensity score matching without replacement and limiting the maximum distance between matches with a caliper. Normalized differences in means are calculated as recommended by [5]. There is no test for statistical significance associated with normalized differences in means but a rule of thumb is that sizes larger than 0.25 can bias simple ordinary least squares regression.

^a^After matching, pre-treatment forest disturbance was “0” for both protected areas and areas outside of protected areas; thus, differences in means could not be calculated.

The estimated treatment effects for Russia range between -0.01 and -0.03 (Table 3). The interpretation is that protected areas reduce the probability that a forested plot is disturbed by as much as -0.03 percentage points over each of the three five-year periods between 1995 (the year after creation of the protected areas) and 2010 (the last year of data). For context, the average five-year probability that a potential control plot was disturbed after 1995 was 0.08% (Table 4). Whether or not the effect of protected areas on forest disturbance is statistically significant or not varies across estimators. When matching with differences in means or cross-sectional regression is estimated, the treatment effect is statistically significant at the 95% level or higher and closer to -0.03 percentage points. With no matching plus fixed effects the estimated treatment effect is smaller, at about -0.02 percentage points, as is the statistical significance, at the 90% level. When matching is used to pre-process the data before fixed effects regression, the treatment effect is not statistically significant. Full regression output for this case can be found in Tables B–D in S2 Table. In Fig 3 we plot the 95% confidence intervals for these treatment effects; again, there is considerable overlap across each estimator.

For calculating the relative effect of Russia-Protected Areas on forest disturbance we divide the average treatment effect (Table 3) by the average forest disturbance in the control sample. For the three estimators that use matching, average forest disturbance in the matched control plots is 0.07 (not reported in the table). Thus, for matching plus difference in means we divide 0.03 by 0.07 to get a relative reduction in forest disturbance of 36% (Fig 3). When fixed effects regression without matching is used, average forest disturbance for all areas outside of parks is 0.08 (Table 4). Dividing the average treatment effect of 0.02 by 0.08 generates a relative reduction in forest disturbance of 23% (Fig 3).

### Comparing Estimators

The two cases we evaluate are similar to many published conservation evaluations of PES and protected areas. In our Ecuador-PES case we have parcel, or household-level sampling units, even though we do not have household-level data, and are able to control for many covariates included in past studies. In our Russia-Protected Areas case we have pixel level information and sample inside and outside of protected areas, controlling for many common observables that control for location and accessibility. Across these two conservation programs, the different sampling and estimation strategies (Fig 1) result in different conclusions about the effectiveness of the conservation policy. One must be cautious, however, in interpreting the reason for the differences in average treatment effects in Table 3 or relative impacts presented in Fig 3. Some estimators differ in more than one aspect, and so it is not clear which aspect explains the difference in estimates. The most telling comparisons are those that differ in only one key aspect, these include (1) matching with differences in means to matching combined with cross-sectional regression; (2) no matching with fixed effects to matching combined with fixed effects; and (3) matching with cross-sectional regression to matching combined with fixed effects.

Considering the first comparison, cross-sectional regression following matching can provide additional control over differences in means if observable covariates are not completely balanced in matching. For both the Ecuador-PES and Russia-Protected Areas cases, the adjustment on observables after matching has little to no effect on the interpretation of conservation impacts. In Ecuador-PES, the statistical significance of the results does not change; in Russia-Protected Areas the statistical significance decreases from the 99% to 95% level. Of course, in other examples and contexts, this additional adjustment on observables could lead to changes in treatment effects, and is generally recommended [5,50,54].

In the second comparison, pre-processing the data with propensity score matching changes the sample used in estimation. Trimming the sample to ensure treatment and control parcels are similar in observable characteristics matters when there is some misspecification of how the observed independent variables drive land cover change, and such misspecification is a common challenge for applied work. Further, since panel fixed effects estimation typically assumes that treatment and control households have a common response to the conservation treatment, this assumption is more plausible in a matched sample than in a sample with very different treatment and control households. Our results suggest that trimming the sample to include only good matches is especially important for Ecuador-PES, increasing the relative impact by 30% in Fig 3 when the average treatment effect increases from 0.31 to 0.42 (Table 3). For protected areas in Russia, the relative impacts in Fig 3 are within 3% of one another, but it decreases enough when the matched sample is used that the estimate changes from 90% significance level to no longer statistically significant. In general, when treatment observations are quite different from controls, matching provides an additional assurance in getting the two groups as similar as possible before panel regression analysis, similar to what has been shown in the case of cross-sectional regression analysis [48–52].

In the comparison of matching combined with cross-sectional versus matching combined with fixed effects regression, the same sample of treatment and control observations are used but the estimators differ in the control of time-invariant unobservables. Thus, we see that explicit control of fixed effects in a panel setting is responsible for differences in conclusions about a conservation programs’ relative effect on forest cover change in Fig 3 ranging from between an increase of 4% (Ecuador-PES) to a decrease of 16% (Russia-protected areas) when time-invariant unobservables are controlled. In the case of Ecuador, the policy implications would be similar between these two estimators, as both estimators come to similar conclusions about statistical significance and size. Thus, in this case the observable covariates do a good job of controlling for confounding factors. In the case of Russia, however, when time-invariant unobservables are controlled the program does not have a statistically significant effect on the probability of forest disturbance. In the Russia case, these time-invariant unobservables are likely related to the hypothesized missing measures for timber value—when the low timber values of protected areas are controlled for with fixed effects the impact estimate is reduced.

The explicit control of fixed effects is the primary advantage to using panel data. The control of fixed effects matters when there is some correlation between the time-invariant unobserved determinants of land cover change, and the location of the conservation program. If time-invariant unobservables are not a concern, estimation under cross-sectional or fixed effects methods will yield similar results. These two conservation programs illustrate that the importance of hidden bias in conservation evaluation will vary. In the Ecuador-PES case, when matching with cross-sectional or matching with fixed effects panel regression is used, the estimated treatment effects are almost identical. This indicates that time-invariant unobservables are not a large concern. In contrast, for the Russia-Protected Areas case, the conclusion of statistically significant conservation impacts does not hold when time-invariant unobservables are controlled (Table 3). In other datasets and examples, the direction of bias from time-invariant unobservables and their importance will also vary. The researcher will have to critically evaluate the likelihood of time invariant unobservables in each case, and the validity of the assumption that treatment effects can be estimated on observable data alone, as is the case with cross-sectional estimation strategies. It is important to re-emphasize as well, that no quasi-experimental design can control for potential time-varying unobservable bias.

## Conclusion

The conservation community is increasingly evaluating if conservation tools are effective and incorporating this information into decision-making. For conservation policies and programs that have a goal of affecting land cover change, the ability to conduct a rigorous quasi-experimental evaluation is bolstered by the release of the Landsat archives [67], the advancement of remote sensing techniques to provide temporally-rich land cover change classifications [68,69], and the publication of free, global datasets of forest cover [46,70]. The number of studies using remote sensing products and quasi-experimental techniques to conduct an impact evaluation is increasing rapidly and the majority of these rely on matching estimators combined with differences in means or cross-sectional regression to estimate treatment effects exclusively on observable covariates. The results from our comparison of estimators across two conservation programs illustrate that estimated treatment effects can vary across estimators, and that in some cases time-invariant unobservables can lead to differences in statistical significance and differences in magnitude of relative impacts. These estimated differences could affect policy lessons and actions such as who should get paid in incentive-based programs, allocation of funding for protected areas, and whether a program should be scaled up or stopped. However, if time-invariant unobservables are not a concern, then estimates from matching combined with cross-sectional regression result in similar policy lessons.

While bias from time-invariant unobservables could be reduced in cross-sectional analyses by collecting more data on plot or parcel characteristics or instrumenting for the conservation program, such data is not always easily available or well measured, and conservation instruments are often far from obvious. An easier solution is often to build better temporal variation with spatial data on land cover change that can reduce the number of assumptions required for identification of conservation program effectiveness. This allows the researcher to control for many of the observable variables already commonly included in evaluation studies—since they do not vary over time—as well as hidden sources of time-invariant bias through the fixed effect, as long as the unit of analysis corresponds with the appropriate decision-making unit. Combining matching to pre-process the dataset with fixed effects regression to control for time-invariant unobservables has also been shown to be one of the most robust strategies for replicating experimental evidence of conservation effectiveness [43]. The identification of conservation impacts with fixed effects relies on two conditions: (1) repeated remote sensing landscape images over time, and (2) temporal variation in the location of conservation programs within the time frame of the estimation sample.

If these two conditions do not hold for a particular conservation policy or program, then matching combined with t-tests or cross-sectional estimation approaches will be the only alternative. Our findings suggest that when these methods are used, they are likely still in range of the average treatment effect estimate that would be obtained from fixed effects regression, as indicated by the overlap in confidence intervals in our two examples in Fig 3. Across both cross-sectional and panel program evaluation methods, researchers should take greater care to emphasize the uncertainty associated with the estimated average treatment effect and report the range of values using the 95% confidence interval, in addition to just reporting the mean effect. Additionally, if researchers are able to hypothesize on the potential sources of bias from time-invariant and time-varying unobservables they can discuss the direction of that bias, and how this would affect estimated treatment effects. These best practices can help move the field of conservation evaluation to more rigorous, and reliable, estimates of the impact of conservation policies and programs on land cover change outcomes, and facilitate synthesis of impacts across multiple studies in systematic reviews and meta-analysis [71].

## Supporting Information

S1 Table Regression Output for Ecuador-PES. (DOCX)

S2 Table Regression Output for Russia-Protected Areas. (DOCX)

## References

[1] Ferraro PJ, Pattanayak SK. Money for nothing? A call for empirical evaluation of biodiversity conservation instruments. PLoS Bio. 2006; 4: e105. 16602825 10.1371/journal.pbio.0040105 PMC1435411

[2] Mickwitz P, Birnbaum M. Key insights on the design of environmental evaluations. In Birnbaum M, Mickwitz P (Editors), Environmental program and policy evaluation: Addressing methodological challenges. New Directions for Evaluation 2009; 122: 105–112.

[3] Ferraro PJ. Counterfactual thinking and impact evaluation in environmental policy. In Birnbaum M. and Mickwitz P (Editors), Environmental program and policy evaluation: Addressing methodological challenges. New Directions for Evaluation 2009; 122: 75–84.

[4] Margoluis R, Stem C, Salafsky N, Brown M. Design alternatives for evaluating the impact of conservation projects. In Birnbaum M, Mickwitz P (Editors), Environmental program and policy evaluation: Addressing methodological challenges. New Directions for Evaluation 2009; 122: 85–96.

[5] Imbens GW, Wooldridge JM. Recent developments in the econometrics of program evaluation. J Econ Lit. 2009; 47: 5–86.

[6] Joppa L, Pfaff A. High and far: Biases in the location of protected areas. PLoS One. 2009; 4: e8273 10.1371/journal.pone.0008273 20011603 PMC2788247

[7] Andam KS, Ferraro PJ, Pfaff A, Sanchez-Azofeifa GA, Robalino JA. Measuring the effectiveness of protected area networks in reducing deforestation. PNAS. 2008; 105(42): 16089–16094. 10.1073/pnas.0800437105 18854414 PMC2567237

[8] Andam KS, Ferraro PJ, Hanauer MM. The effects of protected area systems on ecosystem restoration: a quasi-experimental design to estimate the impact of Costa Rica’s protected area system on forest regrowth. Consv Letters. 2013; 6(5): 317–323.

[9] Beresford AE, Eshiamwata GW, Donald PF, Balmford A, Bertzky B, Brink AB, et al Protection reduces loss of natural land-cover at sites of conservation importance across Africa. PLOS One. 2013; 8(5): e65370 10.1371/journal.pone.0065370 23734249 PMC3667134

[10] Carranza T, Balmford A, Kapos V, Manica A. Protected area effectiveness in reducing conversion in a rapidly vanishing ecosystem: the Brazilian Cerrado. Consv Letters. 2013; 7(3): 216–223.

[11] Carranza T, Manica A, Kapos V, Balmford A. Mismatches between conservation outcomes and management evaluation in protected areas: a case study in the Brazilian Cerrado. Bio Consv. 2014; 173: 10–16.

[12] Ferraro PJ, Hanauer MM. Protecting ecosystems and alleviating poverty with parks and reserves: ‘win-win’ or tradeoffs? Env Res Econ. 2011; 48 (2): 269–286.

[13] Ferraro PJ, Hanauer MM, Miteva DA, Canavire-Bacarreza GJ, Pattanayak SK, Sims KRE. More strictly protected areas are not necessarily more protective: evidence from Bolivia, Costa Rica, Indonesia, and Thailand. Env Res Let. 2013; 8: 1–8.

[14] Gaveau DLA, Epting J, Lyne O, Linkie M, Kumara I, Kanninen M, Leader-Williams N. 2009 Evaluating whether protected areas reduce tropical deforestation in Sumatra. Jour of Biogeo. 2009; 36: 2165–2175.

[15] Gaveau DLA, Curran LM, Paoli GD, Carlson KM, Wells P, Besse-Rimba A, et al Examining protected area effectiveness in Sumatra: importance of regulations governing unprotected lands. Cons Letters. 2012; 5: 142–148.

[16] Gaveau DLA, Kshatriya M, Shell D, Sloan S, Molidena E, Wijaya A, et al Reconciling forest conservation and logging in Indonesian Borneo. PLOS One. 2013; 8(8): e69887 10.1371/journal.pone.0069887 23967062 PMC3743885

[17] Haruna A, Pfaff A, van den Ende S, Joppa L. Evolving protected-area impacts in Panama: impact shifts show that plans require anticipation. Env Res Let. 2014; 9: 1–11.

[18] Joppa L, Pfaff A. Global protected area impacts. Proc Roy Soc. 2011; 278 (1712): 1633–1638. 10.1098/rspb.2010.1713 PMC3081759 21084351

[19] Nelson A, Chomitz KM. Effectiveness of strict vs. multiple use protected areas in reducing tropical forest fires: a global analysis using matching methods. PLoS ONE. 2011; 6: e22722 10.1371/journal.pone.0022722 21857950 PMC3156699

[20] Nolte C, Agrawal A, Silvius KM, Soares-Filho BS. 2013 Governance regime and location influence avoided deforestation success of protected areas in the Brazilian Amazon. PNAS. 2013; 110 (13): 4956–4961. 10.1073/pnas.1214786110 23479648 PMC3612687

[21] Nolte C, Agrawal A. Linking management effectiveness indicators to observed effects of protected areas on fire occurrence in the Amazon rainforest. Cons Bio. 2013; 27(1): 155–165. 10.1111/j.1523-1739.2012.01930.x 23009052

[22] Pfaff A, Robalino J, Sanchez-Azofeifa GA, Andam KS, Ferraro PJ. Park location affects forest protection: Land characteristics cause differences in park impacts across Costa Rica. The B.E. Journal of Economic Analysis and Policy 2009; 9(2): 1–24.20098633

[23] Pfaff A, Robalino J, Lima E, Sandoval C, Herrera LD. 2014 Governance, location and avoided deforestation from protected areas: greater restrictions can have lower impact, due to differences in location. Wor Dev. 2014; 55: 7–20.

[24] Sieber A, Kummerle T, Prishchepov AV, Wendland KJ, Baskin LM, Radeloff VC, Hostert P. Post-Soviet land-use change and the effectiveness of strictly protected areas in European Russia. Rem Sens Env. 2013; 133: 38–51.

[25] Vergara-Asenjo G, Potvin C. Forest protection and tenure status: The key role of indigenous peoples and protected areas in Panama. Glob Env Ch. 2014; 28: 205–215.

[26] Bragina EV, Radeloff VC, Baumann M, Wendland K, Kuemmerle T, Pidgeon AM. Effectiveness of protected areas in the Western Caucasus before and after the transition to post-socialism. Bio Consv. 2015; 184: 456–464.

[27] Wendland KJ, Baumann M, Lewis D, Sieber A, Radeloff V. Protected area effectiveness in European Russia: a post-matching panel data analysis. Land Econ. 2015; 91(1): 149–168.

[28] Clements T, Milner-Gulland EJ. Impact of payments for environmental services and protected areas on local livelihoods and forest conservation in northern Cambodia. Consv Bio. 2014; 1–10. 10.1111/cobi.12423 PMC4312980 25492724

[29] Alix-Garcia JM, Shapiro EN, Sims KRE. Forest conservation and slippage: evidence from Mexico’s national payments for ecosystem services program. Land Econ. 2012; 88(4): 613–638.

[30] Alix-Garcia JM, Sims KRE, Yanez-Pagans P, Radeloff V, Shapiro EN. Only one tree from each seed? Environmental effectiveness and poverty alleviation in programs of payments for ecosystem services. Work Pap Univ Wis Madison. 2014; Available: http://www.aae.wisc.edu/alixgarcia/One%20Tree%20Manuscript%20FINAL.pdf

[31] Arriagada RA, Ferraro PJ, Sills EO, Pattanayak SK, Cordero-Sancho S. Do payments for environmental services affect forest cover? A farm-level evaluation from Costa Rica. Land Econ. 2012; 88(2): 382–399.

[32] Bruggeman D, Meyfroidt P, Lambin EF. Production forests as a conservation tool: Effectiveness of Cameroon’s land use zoning policy. Land Use Pol. 2015; 42: 151–164.

[33] Busch J, Ferretti-Gallon K, Engelmann J, Wright M, Austin KG, Stolle F, et al Reductions in emissions from deforestation from Indonesia’s moratorium on new oil palm, timber, and logging concessions. PNAS. 2014; 10.1073/pnas.1412514112 PMC4321246 25605880

[34] Costedoat S, Corbera E, Ezzine-de-Blas D, Honey-Rosés J, Baylis K, Castillo-Santiago MA. How Effective Are Biodiversity Conservation Payments in Mexico? PLoS ONE. 2015; 10(3): e0119881 10.1371/journal.pone.0119881 25807118 PMC4373862

[35] Honey-Roses J, Baylis K, Isabel Ramirez M. A spatially explicit estimate of avoided forest loss. Cons Bio. 2011; 25(5): 1032–1043. 10.1111/j.1523-1739.2011.01729.x 21902720

[36] Robalino J, Pfaff A. 2013 Ecopayments and deforestation in Costa Rica: A nationwide analysis of PSA’s initial years. Land Econ. 2013; 89(3): 432–448.

[37] Scullion JJ, Thomas CW, Vogt KA, Perez-Maqueo O, Logsdon MG. Evaluating the environmental impact of payments for ecosystem services in Coatepec (Mexico) using remote sensing and on-site interviews. Env Cons. 2011; 38(04): 426–434.

[38] Scullion JJ, Vogt KA, Sienkiewicz A, Gmur SJ, Trujillo C. Assessing the influence of land-cover change and conflicting land-use authorizations on ecosystem conversion on the forest frontier of Madre do Dios, Peru. Bio Cons. 2014; 171: 247–258.

[39] Pattanayak SK, Wunder S, Ferraro PJ. Show Me the Money: Do Payments Supply Environmental Services in Developing Countries? Rev Env Econ Pol. 2010; 4(2): 254–274.

[40] Miteva DA, Pattanayak SK, Ferraro PJ. Evaluation of biodiversity policy instruments: what works and what doesn’t? Ox Rev Econ Pol. 2012; 28(1): 69–92.

[41] Samii C, Lisiecki M, Kulkarni P, Paler L, Chavis L. Effects of payment for environmental services (PES) on deforestation and poverty in low and middle income countries: a systematic review. CEE 13-015b. Collaboration for Environmental Evidence. 2014.

[42] Angrist JD, Pischke JS. Mostly Harmless Econometrics: An Empiricist’s Companion. Princeton University Press; 2009.

[43] Ferraro PJ, Miranda JJ. Panel data designs and estimators as substitutes for randomized controlled trials in the evaluation of social programs. Work Pap Georgia State Univ Atlanta. 2014; Available: http://www2.gsu.edu/~wwwcec/docs/Ferraro%20and%20Miranda%20Panel%20Data%20Rep%20POST.pdf

[44] Joppa L, Pfaff A. Reassessing the forest impacts of protection: The challenge of nonrandom location and a corrective method. Ann NY Acad Sci. 2010; 1185: 135–149. 10.1111/j.1749-6632.2009.05162.x 20146766

[45] Blackman A. Evaluating forest conservation policies in developing countries using remote sensing data: An introduction and practical guide. For Policy & Econ. 2013; 34: 1–16.

[46] Ferraro PJ, Hanauer MM. Advances in measuring the environmental and social impacts of environmental programs. Ann Rev of Env and Res. 2014; 39: 485–517.

[47] Hansen MC, Potapov PV, Moore R, Hancher M, Turubanova SA, Tyukavina A, et al High-resolution global maps of 21st-century forest cover change. Science. 2013; 342 (6160): 850–853. 10.1126/science.1244693 24233722

[48] Rubin D. Matching to Remove Bias in Observational Studies. Biometrics. 1973; 29: 159–183.

[49] Rubin D. The Use of Matched Sampling and Regression Adjustments to Remove Bias in Observational Studies. Biometrics. 1973; 29: 185–203.

[50] Rubin D. Using Multivariate Matched Sampling and Regression Adjustment to Control Bias in Observational Studies, J AM Stat Assoc. 1979; 74: 318–328.

[51] Rosenbaum P. Optimal Matching in Observational Studies. J Am Stat Assoc. 1989; 84: 1024–1032.

[52] Imbens, GW. Matching methods in practice: Three examples. NBER Working Paper 19959; 2014.

[53] Abadie A, Drukker D, Herr JL, Imbens G. Implementing Matching Estimators for Average Treatment Effects in STATA. The Stata Journal 2003; 4(3): 290–311.

[54] Guo S, Fraser MW. Propensity score analysis: statistical methods and applications. Sage Publications, Washington, D.C.; 2010

[55] de Koning F, Aguinaga M, Bravo M, Chiu M, Lascano M, Lozada T, Suarez L. Bridging the gap between forest conservation and poverty alleviation: the Ecuadorian Socio Bosque program. Env Sci & Pol. 2011; 14(5): 531–542.

[56] Arriagada RA, Sills EO, Pattanayak SK, Ferraro PJ. Combining qualitative and quantitative methods to evaluate participation in Costa Rica’s Program of payments for environmental services. J Sustain Forest. 2009; 28: 343e367.

[57] Zbinden S, Lee DR. Paying for environmental services: an analysis of participation in Costa Rica’s PSA program. Wor Dev. 2005; 33(2): 255–272.

[58] Wells MP, Williams MD. Russia’s protected areas in transition: The impacts of perestroika, economic reform and the move towards democracy. Ambio 1998; 27: 198–206.

[59] Radeloff VC, Beaudry F, Brooks TM, Butsic V, Dubinin M, Kuemmerle T, Pidgeon AM. Hot moments for biodiversity conservation. Cons Let. 2013; 6: 58–65.

[60] Potapov P, Turubanova S, Hansen MC. Regional-Scale Boreal Forest Monitoring Using Landsat Data Composites: First Results for European Russia. Rem Sens Env. 2011; 115: 548–61.

[61] Baumann M, Ozdogan M, Kuemmerle T, Wendland KJ, Esipova E, Radeloff VC. Using the Landsat record to detect forest-cover changes during and after the collapse of the Soviet Union in the temperate zone of European Russia. Rem Sens of Env. 2012; 124: 174–184.

[62] Wendland KJ, Lewis D, Alix-Garcia J, Ozdogan M, Baumann M, Radeloff V. Regional- and district-level drivers of forest disturbance in Post-Soviet Russia. Glob Env Ch. 2011; 21(4): 1290–1300.

[63] Wooldridge JM. 2010 Econometric analysis of cross section and panel data. MIT Press; 2010.

[64] Rubin D. Matched Sampling for Causal Effects. Cambridge University Press, New York; 2006.

[65] Cameron CA, Trivedi PK. Microeconometrics: Methods and Applications. Cambridge University Press, New York; 2005.

[66] Goward S, Irons J, Franks S, Arvidson T, Williams D, Faundeen J. Historical record of Landsat global coverage: Mission operations, NSLRSDA, and international cooperator stations. Photo Eng Rem Sens. 2006; 72: 1155–1169.

[67] Huang C, Song K, Kim S, Townshend JRG, Davis P, Masek J. Goward SN. Use of a dark object concept and support vector machines to automate forest cover change analysis. Rem Sens of Env. 2008; 112: 970–985.

[68] Huang C, Goward SN, Masek JG, Gao F, Vermote EF, Thomas N, et al Development of time series stacks of Landsat images for reconstructing forest disturbance history. Int J Dig Ear. 2009; 2: 195–218.

[69] Kim DH, Sexton JO, Noojipady P, Huang C, Anand A, Channan S, et al Global, Landsat-based forest-cover change from 1990 to 2000. Rem Sens Env. 2014; 155:178–193.

[70] Kim DH, Sexton JO, Noojipady P, Huang C, Anand A, Channan S, et al Global, Landsat-based forest-cover change from 1990 to 2000. Rem Sens Env. 2014; 155:178–193.

[71] Pullin AS, Knight TM. Effectiveness in conservation practice: pointers from medicine and public health. Cons Bio 2001; 15: 50–54.

