# Supplementary material for: Estimating the Counterfactual Impact of Conservation Programs on Land Cover Outcomes: The Role of Matching and Panel Regression Techniques
Source: PLoS One. 2015 Oct 26;10(10):e0141380. doi: 10.1371/journal.pone.0141380 (PMC4621053; doi:10.1371/journal.pone.0141380)
Supplement: S1 Table — (DOCX) [file pone.0141380.s001.docx]

**S1 Table. Regression output for Ecuador-PES.**

***Table A. Estimating determinants of participation in Ecuador-PES program with logit model (marginal effects presented) for propensity score matching***

| **Outcome variable: Participation in PES program (1=PES, 0=No PES)** | |
| --- | --- |
| **Variable** | **Marginal effects**  **(Std Error)** |
| Baseline Deforestation (2004-2006)^1^ | -0.02  (0.01) |
| Size of parcel (sq km) | 0.14***  (0.05) |
| Distance to major town (km) | 0.01***  (0.003) |
| Distance to major road (km) | 0.02***  (0.005) |
| Distance to closest river (km) | -0.01*  (0.004) |
| Distance to closest oil well (km) | 0.002  (0.005) |
| *Observations* | *513* |
| *Correctly classified* | *88%* |
| *Wald Chi2* | *47.33**** |

**p<0.1; **p<0.05; ***p<0.01*

*^1^ This shows 2004-2006 baseline deforestation, which is used in PSM to create the matched sample. In Fixed Effects regression, the baseline deforestation used in the match is dropped, thus, Fixed Effects is estimated for the years 2007-2010. To ensure the samples used are comparable we also use 2004-2006 to create the sample for PSM combined with difference in means and PSM with cross-sectional regression.*

***Table B. Post-matching linear cross-sectional regression of impact of Ecuador-PES program on deforestation in 2011-2013***

| **Outcome variable: Average annual deforestation rate between 2011-2013** | |
| --- | --- |
| **Variable** | **Coefficient**  **(Std Error)** |
| PES dummy | -0.40***  (0.13) |
| Baseline Deforestation (2007-2010)^1^ | 0.27***  (0.05) |
| Size of parcel (sq km) | -0.06  (0.14) |
| Distance to major town (km) | -0.01  (0.02) |
| Distance to major road (km) | -0.03  (0.03) |
| Distance to closest river (km) | -0.02  (0.02) |
| Distance to closest oil well (km) | 0.04  (0.05) |
| *Observations* | *112* |
| *R^2^* | *0.19* |
| *F-test* | *9.80**** |

**p<0.1; **p<0.05; ***p<0.01*

*^1^ This controls for 2007-2010 baseline deforestation, which is similar to what the linear fixed effects estimator controls for, after matching the sample based on average 2004-2006 deforestation (see S.1.).*

***Table C. Linear fixed effects panel regression of impact of Ecuador-PES program on deforestation in 2011-2013 using full sample (no matching)***

| **Outcome variable: Average annual deforestation rate between 2011-2013** | |
| --- | --- |
| **Variable** | **Coefficient**  **(Std Error)** |
| PES dummy | -0.30**  (0.12) |
| 2008 Year dummy | 0.08  (0.25) |
| 2009 Year dummy | -0.19  (0.16) |
| 2010 Year dummy | -0.14  (0.14) |
| 2011 Year dummy | -0.15  (0.17) |
| 2012 Year dummy | 0.15  (0.18) |
| 2013 Year dummy | 0.15  (0.11) |
| *Observations* | *3,591* |
| *Within R^2^* | *0.01* |
| *F-test* | *3.92**** |

**p<0.1; **p<0.05; ***p<0.01*

***Table D. Matching combined with linear fixed effects panel regression of impact of Ecuador-PES program on deforestation in 2011-2013***

| **Outcome variable: Average annual deforestation rate between 2011-2013** | |
| --- | --- |
| **Variable** | **Coefficient**  **(Std Error)** |
| PES dummy | -0.42***  (0.14) |
| 2008 Year dummy | 0.26*  (0.14) |
| 2009 Year dummy | -0.04  (0.04) |
| 2010 Year dummy | 0.22  (0.18) |
| 2011 Year dummy | 0.16  (0.13) |
| 2012 Year dummy | 0.46**  (0.17) |
| 2013 Year dummy | 0.34**  (0.08) |
| *Observations* | *784* |
| *Within R^2^* | *0.03* |
| *F-test* | *5.11**** |

**p<0.1; **p<0.05; ***p<0.01*
