# Supplementary material for: Estimating the Counterfactual Impact of Conservation Programs on Land Cover Outcomes: The Role of Matching and Panel Regression Techniques
Source: PLoS One. 2015 Oct 26;10(10):e0141380. doi: 10.1371/journal.pone.0141380 (PMC4621053; doi:10.1371/journal.pone.0141380)
Supplement: S2 Table — (DOCX) [file pone.0141380.s002.docx]

**S2 Table. Regression output for Russia-Protected Areas.**

***Table A. Estimating determinants of protected area location in Russia with logit model (marginal effects presented) for propensity score matching***

| **Outcome variable: Protected area (1=protected, 0=not protected)** | |
| --- | --- |
| **Variable** | **Marginal effects**  **(Std Error)** |
| Distance to forest edge (1990) | 0.17*  (0.09) |
| Distance to major town (km) | -0.001  (0.05) |
| Distance to capital Moscow (km) | -0.001**  (0.0003) |
| Distance to major road (km) | 0.03  (0.03) |
| Elevation | -0.003***  (0.001) |
| Slope | 0.01**  (0.007) |
| *Observations* | *27,032* |
| *Correctly classified* | *84%* |
| *Wald Chi2* | *183.17**** |

**p<0.1; **p<0.05; ***p<0.01*

***Table B. Post-matching linear^1^ cross-sectional regression of impact of Russia-protected areas on forest disturbance over 1995-2010***

| **Outcome variable: Average forest disturbance between 1995-2010** | |
| --- | --- |
| **Variable** | **Coefficient**  **(Std Error)** |
| Protected area dummy | -0.03**  (0.01) |
| Distance to forest edge (1990) | -0.01  (0.02) |
| Distance to major town (km) | -0.0001  (0.001) |
| Distance to capital Moscow (km) | 0.00001  (0.00001) |
| Distance to major road (km) | 0.004  (0.002) |
| Elevation | -0.0006*  (0.0003) |
| Slope | 0.007**  (0.004) |
| *Observations* | *9,204* |
| *R^2^* | *0.02* |
| *F-test* | *10.00**** |

**p<0.1; **p<0.05; ***p<0.01*

*^1^ We estimate a linear cross-sectional regression since we are interested in marginal effects, and it has been shown that under similar identifying assumptions, linear estimation generates similar marginal effects as non-linear methods [54]. For comparison, the marginal effects after estimating the same equation with logistic regression are also -0.03, significant at the 95% level.*

***Table C. Linear fixed effects panel regression of impact of Russia-protected areas on forest disturbance over 1995-2010 (no matching)***

| **Outcome variable: Average forest disturbance between 1995-2010** | |
| --- | --- |
| **Variable** | **Coefficient**  **(Std Error)** |
| Protected area dummy | -0.018*  (0.011) |
| Distance to forest edge | -0.013**  (0.005) |
| 1995-2000 dummy | 0.026***  (0.004) |
| 2000-2005 dummy | 0.051***  (0.006) |
| 2005-2010 dummy | 0.043**  (0.004) |
| *Observations* | *106,950* |
| *Within R^2^* | *0.03* |
| *F-test* | *25.57**** |

**p<0.1; **p<0.05; ***p<0.01*

***Table D. Matching combined with linear fixed effects panel regression of impact of Russia-protected areas on forest disturbance over 1995-2010***

| **Outcome variable: Average forest disturbance between 1995-2010** | |
| --- | --- |
| **Variable** | **Coefficient**  **(Std Error)** |
| Protected area dummy | -0.014  (0.011) |
| Distance to forest edge | -0.010**  (0.005) |
| 1995-2000 dummy | 0.020***  (0.006) |
| 2000-2005 dummy | 0.051***  (0.010) |
| 2005-2010 dummy | 0.035***  (0.005) |
| *Observations* | *36,217* |
| *Within R^2^* | *0.03* |
| *F-test* | *21.77**** |

**p<0.1; **p<0.05; ***p<0.01*
